# Prevalence and associated factors of thyroid nodules in a health examination cohort: an ultrasound-based cross-sectional study using electronic health records

**DOI:** 10.3389/fpubh.2026.1752140

**Published:** 2026-03-04

**Authors:** Lele Meng, Xueying Zhang

**Affiliations:** 1 Health Management Center of the First Affiliated Hospital of Henan Polytechnic University (Jiaozuo Second People’s Hospital), Jiaozuo, Henan, China; 2 Department of Dermatology, Jiaozuo City Hospital of Traditional Chinese Medicine, Jiaozuo, Henan, China

**Keywords:** cross-sectional study, iodine, prevalence, risk factors, thyroid function, thyroid nodules

## Abstract

**Objectives:**

This large-scale cross-sectional study utilized electronic health records (EHRs) to determine the prevalence, sonographic characteristics, and independent correlates of thyroid nodules in a Chinese health examination cohort, with comprehensive adjustment for iodine status, thyroid function, and autoantibodies.

**Methods:**

We analyzed data from 12,468 adults undergoing routine check-ups in 2024. Thyroid nodules were assessed via ultrasonography and classified using TI-RADS. Multivariable logistic regression identified factors independently associated with nodule presence.

**Results:**

The prevalence of thyroid nodules was 35.36%, with the vast majority (96.01%) being benign or probably benign (TI-RADS 2–3). Independent correlates included female sex (OR = 1.65, 95% CI:1.51–1.80), older age (per 10-year increment, OR = 1.55, 95% CI:1.47–1.64), family history of thyroid disease (OR = 2.31, 95% CI:2.04–2.61), insufficient iodine intake (OR = 1.38, 95% CI:1.23–1.55), obesity (OR = 1.35, 95% CI:1.21–1.50), abnormal TSH (OR = 1.32, 95% CI:1.19–1.46), and TPOAb positivity (OR = 1.28, 95% CI:1.13–1.45). Regular moderate-to-vigorous physical activity was protective (OR = 0.73, 95% CI:0.66–0.81).

**Conclusion:**

Thyroid nodules are highly prevalent in this population. We identified a profile of both non-modifiable (e.g., sex, age) and modifiable (e.g., iodine nutrition, obesity, physical activity) correlates. These findings highlight targets for preventive health strategies, though future prospective studies in the general population are warranted to confirm causality.

## 1 Introduction

Thyroid nodules are among the most prevalent endocrine disorders globally (1). In China, ultrasound-based detection rates vary widely—from 20% to 76%—reflecting regional disparities in iodine nutrition, metabolic profiles, genetic susceptibility, and screening accessibility (2, 3). While only 5%–15% of nodules are malignant, their high prevalence poses a considerable clinical and public health burden, contributing to patient anxiety and increased healthcare utilization (4).

Jiaozuo, an industrial city in northwestern Henan Province, presents a distinctive setting for thyroid health research. As an inland region, its population may exhibit iodine nutrition patterns that contrast with those of coastal areas, while local industrial exposures and dietary habits—such as high salt intake—may further modulate thyroid disease risk (5–7). Previous studies in this region have often relied on self-reported data, which are susceptible to recall bias, and have inadequately controlled for crucial confounders including iodine status, thyroid function, and autoantibodies, thereby limiting the validity of their conclusions. In contrast, electronic health records (EHRs) provide an objective and integrated data source, combining clinical, laboratory, and historical information to minimize measurement bias and support more rigorous adjustment for confounders. It is important to note, however, that health check-up populations are prone to the “healthy worker effect,” wherein participants typically demonstrate greater health awareness and socioeconomic advantage compared to the general population, thus restricting the external validity of findings. Moreover, given the multifactorial etiology of thyroid nodules—influenced by genetic, environmental, and endocrine factors—omitting key determinants such as iodine intake, TSH levels, and autoimmunity may introduce residual confounding and undermine the identification of true independent correlates (8).

To address the need for integrated multi-dimensional evidence, this study utilized a large-scale electronic health record (EHR) database from a health examination cohort. Its core contribution is the simultaneous assessment of iodine nutrition, thyroid function, autoimmunity, and lifestyle factors within a unified analytical framework, allowing for comprehensive adjustment via structured EHR data. This approach extends beyond prior studies that often examined these correlates in isolation. For example, whereas recent work by Weng et al. focused primarily on metabolic risk factors (9), our analysis jointly evaluates endocrine, immune, and behavioral dimensions. We interpret findings within the context of this health-seeking population to ensure methodological rigor, with the aim of informing targeted thyroid health management in similar clinical settings.

## 2 Materials and methods

### 2.1 Study design and population

This cross-sectional study consecutively enrolled adults who underwent routine health examinations at the Health Management Center of Jiaozuo Second People’s Hospital between January 1 and December 31, 2024. The study protocol received approval from the Medical Ethics Committee of Jiaozuo Second People’s Hospital (Approval No: KY2025-11-010) and was conducted in accordance with the principles of the Declaration of Helsinki. Due to the retrospective design, which relied exclusively on anonymized electronic health record (EHR) data, the requirement for informed consent was waived.

Eligible participants were required to be aged 18 years or older and to have completed a standardized check-up protocol that included thyroid ultrasonography, anthropometric measurements, and essential laboratory tests—specifically thyroid function, thyroid autoantibodies, and urinary iodine concentration. Additionally, individuals were only included if their EHRs contained complete data on demographic characteristics, medical history, and lifestyle factors, defined as having less than 20% missing information across these domains. Key exclusion criteria comprised a history of thyroid surgery, radioiodine therapy, or neck radiotherapy; a prior pathological diagnosis of thyroid nodules (e.g., via fine-needle aspiration or surgery); current pregnancy or lactation; severe hepatic or renal dysfunction (Child-Pugh class C or eGFR <30 mL/min/1.73m^2^); and current use of medications known to affect thyroid function, such as levothyroxine, methimazole, lithium, or amiodarone.

After applying these criteria, a total of 12,468 participants were included in the final analysis. An *a priori* sample size calculation was performed to ensure statistical robustness. Based on an anticipated thyroid nodule prevalence of 35% (3), with *α* = 0.05, *β* = 0.2 (power = 80%), and a minimum detectable odds ratio of 1.5 for key exposures such as insufficient iodine intake, a minimum of 10,800 participants was required. The final sample size of 12,468 exceeded this threshold, ensuring sufficient power to detect clinically meaningful associations.

### 2.2 Data source and variable definition

All study data were systematically extracted from the hospital’s integrated electronic health record (EHR) system, which consolidates standardized information from health examinations, laboratory testing, medication records, and clinical diagnoses into a unified platform. The primary outcome of this study was the presence of thyroid nodules, defined as the sonographic identification of one or more focal lesions with a diameter of 3 mm or greater. All detected nodules were classified according to the Chinese Thyroid Imaging Reporting and Data System (TI-RADS) (1). Under this system, TI-RADS 2 indicates benign nodules (e.g., simple cysts or spongiform nodules); TI-RADS 3 indicates probably benign nodules with a malignancy risk below 2%; TI-RADS 4A reflects mildly suspicious nodules (malignancy risk 2%–10%); and TI-RADS 4B-5 denotes highly suspicious nodules with a malignancy risk exceeding 10%. For analytical purposes, nodules categorized as TI-RADS 4A or higher were collectively defined as “suspicious malignant nodules.”

A comprehensive set of explanatory variables was pre-specified based on clinical relevance and prior literature. Demographic variables included sex, age (analyzed continuously and categorized into <30, 30–39, 40–49, and ≥50 years for descriptive summaries), educational attainment (primary school or below, junior high school, high school/technical secondary school, college or above), and occupation type (e.g., government/institutional employee, enterprise employee, self-employed, retired, other). Iodine nutrition status was assessed via urinary iodine concentration (UIC), measured using arsenic-cerium catalytic spectrophotometry, and classified as insufficient (<100 μg/L), adequate (100–299 μg/L), or excessive (≥300 μg/L). Key metabolic indicators comprised body mass index (BMI), calculated as weight in kilograms divided by height in meters squared and categorized per Chinese guidelines as underweight (<18.5 kg/m^2^), normal (18.5–23.9 kg/m^2^), overweight (24.0–27.9 kg/m^2^), or obese (≥28.0 kg/m^2^). Hypertension was defined as systolic blood pressure ≥140 mmHg and/or diastolic blood pressure ≥90 mmHg or a prior physician diagnosis; diabetes was defined as fasting blood glucose ≥7.0 mmol/L or a previous clinical diagnosis.

Thyroid function was evaluated using serum levels of thyroid-stimulating hormone (TSH; reference range: 0.27–4.2 mIU/L), free triiodothyronine (FT3; 3.1–6.8 pmol/L), and free thyroxine (FT4; 12.0–22.0 pmol/L). Abnormal TSH was defined as values outside the reference interval, encompassing both subclinical and overt thyroid dysfunction. Thyroid autoimmunity was assessed via thyroid peroxidase antibody (TPOAb; positive ≥34 IU/mL) and thyroglobulin antibody (TgAb; positive ≥115 IU/mL). Autoimmune thyroiditis (AIT) was defined as seropositivity for TPOAb and/or TgAb, while a clinical history of thyroiditis (e.g., Hashimoto’s thyroiditis, subacute thyroiditis) was ascertained from EHR documentation (10–12).

Lifestyle variables included smoking status (never vs. ever—the latter defined as having smoked for at least 1 year or having quit within the past year), alcohol consumption (never vs. regular drinking, the latter defined as consuming two or more alcoholic drinks per week for five or more years), and physical activity level, classified as either <150 min or ≥150 min per week of moderate-to-vigorous intensity exercise (≥3 METs) (13). Relevant medical history variables included family history of thyroid disease among first- or second-degree relatives and history of non-neck radiation exposure, encompassing both medical (e.g., CT or MRI scans) and occupational sources.

### 2.3 Ultrasonographic examination and quality control

Thyroid ultrasonography was uniformly performed using a Logiq E9 system (GE Healthcare, USA) equipped with a 7.5–10 MHz linear array transducer. All scans were conducted by certified physicians with at least 5 years of specialized experience in thyroid imaging, adhering to a standardized protocol to systematically evaluate nodule characteristics including size, margin, shape, internal echogenicity, presence of calcifications, and vascularity in accordance with established guidelines (1). To ensure diagnostic consistency, inter-observer agreement was formally assessed through independent re-evaluation of a randomly selected 10% of participants (*n* = 1,247) by a second senior physician, yielding a Kappa statistic of 0.89 (95% CI, 0.86–0.92), which reflects excellent reproducibility.

A multi-layered quality control framework was implemented throughout the study. During the data extraction phase, two trained researchers independently abstracted information from the electronic health records, with any discrepancies adjudicated by a third senior researcher. Double data entry was employed for key variables—including TI-RADS classification, TSH levels, TPOAb status, and medication history—to minimize transcription errors. The overall rate of missing data was 3.2%, primarily affecting lifestyle-related variables. To address this, multiple imputation using the MICE algorithm was applied, creating five imputed datasets with 10 iterations. The imputation model incorporated all explanatory and outcome variables to preserve statistical validity and reduce bias (14). Distributions of key variables were compared before and after imputation, confirming the robustness of the imputation process.

Laboratory measurements—including urinary iodine concentration, thyroid function tests, and autoantibody assays—were performed in the hospital’s accredited clinical laboratory, which maintains rigorous internal quality control procedures and participates in external quality assessment schemes to ensure analytical accuracy. Furthermore, all sonographers involved in the study received uniform training on the application of the TI-RADS classification criteria prior to the study’s initiation, thereby standardizing diagnostic assessments across operators.

### 2.4 Statistical analysis

All statistical analyses were conducted using SPSS Statistics 26.0 (IBM Corp., USA) and R 4.2.1 (R Foundation for Statistical Computing). Continuous variables are summarized as mean±standard deviation if normally distributed, or as median with interquartile range otherwise; group comparisons were performed using Student’s *t*-test, ANOVA, or the Kruskal–Wallis test as appropriate. Categorical variables are presented as frequencies with percentages, and compared using the chi-square test or Fisher’s exact test for small cell counts.

To identify factors independently associated with thyroid nodules, multivariable logistic regression was applied. Potential collinearity among independent variables was carefully considered during model construction. In particular, variables with known biological correlations (e.g., urinary iodine, TSH, and TPOAb) were rigorously evaluated. Given their irreplaceable clinical and pathophysiological significance, all were retained in the final model. For missing data (overall missing rate 3.2%), we performed multiple imputation using the Multivariate Imputation by Chained Equations (MICE) algorithm. Five imputed datasets were created with 10 iterations to ensure convergence, and the imputation model included all analytical variables. A comparison of key variable distributions before and after imputation confirmed the robustness of the procedure (14). Variable selection was conducted *a priori* based on established biological plausibility and previous literature, and included sex, age (as a continuous variable), iodine nutrition status, BMI category, TSH status (normal/abnormal), TPOAb status (negative/positive), history of thyroiditis, physical activity level, family history of thyroid disease, and history of non-neck radiation exposure. Results are reported as adjusted odds ratios (ORs) with corresponding 95% confidence intervals. Owing to the limited number of cases classified as suspicious malignant nodules (*n* = 176), correlates for this subgroup were analyzed descriptively and presented as crude ORs without multivariable adjustment.

A two-tailed *p*-value <0.05 was considered statistically significant. In line with the cross-sectional design of the study, all results are interpreted as statistical associations rather than evidence of causation, and caution is exercised to avoid overinterpretation.

## 3 Results

### 3.1 Baseline characteristics of the study population

The final analytical cohort comprised 12,468 adults, of whom 5,823 (46.70%) were male and 6,645 (53.30%) were female, with a mean age of 42.3 ± 11.5 years. The majority of participants exhibited adequate iodine nutrition (68.23%) and fell within the normal body mass index range (41.25%). Thyroid function, as reflected by TSH levels, was within the normal range in 79.36% of the population. Evidence of thyroid autoimmunity—defined as positivity for TPOAb and/or TgAb—was present in 12.86% of subjects, while 8.76% had a documented clinical history of thyroiditis. A family history of thyroid disease was reported by 1,205 individuals (9.66%), and 3,740 participants (29.99%) engaged regularly in moderate-to-vigorous physical activity, as defined by ≥150 min per week. Consistent with the study’s exclusion criteria, no participants were using medications known to influence thyroid function at the time of assessment. The overall profile reflects a generally middle-aged health-seeking population with a low prevalence of major thyroid-related comorbidities. A comprehensive summary of baseline characteristics, stratified by thyroid nodule status, is presented in Table 1.

**Table 1:** Baseline characteristics of the study population (*n* = 12,468).

Characteristic	Category	Number (*n*)	Proportion (%)
Sex	Male	5,823	46.70
	Female	6,645	53.30
Age (years)	<30	1,892	15.18
	30 ~ 39	3,215	25.79
	40 ~ 49	4,103	32.91
	≥50	3,258	26.13
Education level	Primary school or below	865	6.94
	Junior high school	2,677	21.47
	High school / secondary vocational	4,231	34.09
	College or above	4,695	37.60
Occupation	Government institution	2,891	23.20
	Enterprise employee	4,562	36.59
	Freelancer	1,683	13.50
	Retired	1,985	15.92
	Other	1,347	10.80
Iodine nutrition status	Insufficient (<100 μg/L)	2,231	17.89
	Adequate (100 ~ 299 μg/L)	8,507	68.23
	Excessive (≥300 μg/L)	1,730	13.87
BMI (kg/m^2^)	Underweight (<18.5)	523	4.20
	Normal (18.5 ~ 23.9)	5,140	41.25
	Overweight (24.0 ~ 27.9)	4,765	38.22
	Obese (≥28.0)	2,040	16.33
TSH status	Normal	9,895	79.36
	Abnormal	2,573	20.64
TPOAb status	Negative	10,871	87.14
	Positive	1,597	12.86
History of thyroiditis	No	11,378	91.24
	Yes	1,090	8.76
Smoking	No	10,485	84.09
	Yes	1,983	15.91
Alcohol consumption	No	10,363	83.11
	Yes	2,105	16.89
Weekly physical activity	<150 min	8,728	70.01
	≥150 min	3,740	29.99
Family history of thyroid disease	No	11,263	90.34
	Yes	1,205	9.66
Non-neck radiation exposure	No	10,893	87.37
	Yes	1,575	12.63
Hypertension	No	9,577	76.80
	Yes	2,891	23.20
Diabetes	No	11,436	91.72
	Yes	1,032	8.28

### 3.2 Prevalence and sonographic characteristics of thyroid nodules

Thyroid nodules were sonographically identified in 4,409 participants, yielding an overall prevalence of 35.36% within this health examination cohort. Among the detected nodules, the vast majority (96.01%) were classified as either benign or probably benign: 2,812 (63.78%) as TI-RADS 2 (benign) and 1,421 (32.23%) as TI-RADS 3 (probably benign). A smaller proportion of nodules exhibited features warranting higher clinical suspicion, with 156 (3.54%) categorized as TI-RADS 4A (mildly suspicious) and 20 (0.45%) as TI-RADS 4B–5 (highly suspicious). Collectively, nodules considered suspicious for malignancy (defined as TI-RADS 4A or higher) accounted for 3.99% of all detected nodules. This distribution underscores that while thyroid nodules are a common sonographic finding in this population, the overwhelming majority display imaging features associated with a low risk of malignancy.

### 3.3 Univariate analysis of factors associated with thyroid nodules

In the univariate analysis, multiple demographic, metabolic, and lifestyle factors demonstrated significant associations with the presence of thyroid nodules. Specifically, female sex, advanced age, insufficient or excessive iodine intake, obesity (BMI ≥ 28 kg/m^2^), abnormal TSH levels, positive TPOAb status, history of thyroiditis, smoking, alcohol consumption, physical inactivity, family history of thyroid disease, and history of non-neck radiation exposure were all significantly associated with a higher prevalence of thyroid nodules (all *p* < 0.05). In contrast, neither hypertension nor diabetes showed a statistically significant association with nodule presence in this initial analysis (both *p* > 0.05; see Table 2). These findings not only align with previously reported risk profiles but also support the inclusion of these significant variables in the subsequent multivariable regression model to identify independent correlates.

**Table 2:** Univariate analysis of factors associated with thyroid nodules (*n*, %).

Characteristic	Category	Total (*n*)	Nodules detected (*n*)	Detection rate (%)	*χ*^2^ value	*p* value
Sex	Male	5,823	1,610	27.65	158.23	<0.001
	Female	6,645	2,799	42.17		
Age (years)	<30	1,892	346	18.29	326.57	<0.001
	30 ~ 39	3,215	951	29.58		
	40 ~ 49	4,103	1,589	38.73		
	≥50	3,258	1,596	48.96		
Iodine nutrition status	Insufficient	2,231	921	41.28	67.89	<0.001
	Adequate	8,507	2,863	33.66		
	Excessive	1,730	625	36.13		
BMI (kg/m^2^)	Underweight	523	157	30.02	45.32	<0.001
	Normal	5,140	1,621	31.54		
	Overweight	4,765	1,723	36.16		
	Obese	2,040	908	44.51		
TSH status	Normal	9,895	3,265	33.00	38.76	<0.001
	Abnormal	2,573	1,144	44.46		
TPOAb status	Negative	10,871	3,698	34.02	49.83	<0.001
	Positive	1,597	711	44.52		
History of thyroiditis	No	11,378	3,912	34.39	28.57	<0.001
	Yes	1,090	497	45.60		
Physical activity	<150 min	8,728	3,362	38.51	67.32	<0.001
	≥150 min	3,740	1,047	28.02		
Family history of thyroid disease	No	11,263	3,706	32.81	243.69	<0.001
	Yes	1,205	708	58.76		
Non-neck radiation exposure	No	10,893	3,761	34.53	18.45	<0.001
	Yes	1,575	648	41.14		

### 3.4 Multivariate analysis of independent correlates of thyroid nodules

Multivariable logistic regression, adjusted for key demographic, metabolic, and immunological confounders, identified several factors independently associated with thyroid nodule presence. Notably, the stability of the coefficient estimates and the absence of inflated standard errors in the final model indicate that collinearity—including the anticipated biological correlation between variables such as TSH and TPOAb status—did not materially threaten the reliability or interpretability of the odds ratios presented below.

Female sex was significantly associated with increased odds of nodules (OR = 1.65, 95% CI: 1.51–1.80), as was advancing age, with each 10-year increase corresponding to an OR of 1.55 (95% CI, 1.47–1.64). Modifiable metabolic and endocrine factors also demonstrated significant associations: insufficient iodine intake (OR = 1.38, 95% CI: 1.23–1.55), obesity (OR = 1.35, 95% CI: 1.21–1.50), abnormal TSH levels (OR = 1.32, 95% CI: 1.19–1.46), and TPOAb positivity (OR = 1.28, 95% CI: 1.13–1.45) were each independently correlated with higher nodule prevalence. A strong association was observed among participants with a family history of thyroid disease (OR = 2.31, 95% CI: 2.04–2.61), while a history of non-neck radiation exposure was associated with more modestly elevated odds (OR = 1.17, 95% CI: 1.05–1.31). In contrast, regular moderate-to-vigorous physical activity emerged as a significant protective factor (OR = 0.73, 95% CI: 0.66–0.81). Several factors that were significant in univariate analysis—including history of thyroiditis, smoking, alcohol consumption, educational attainment, and occupation—did not retain independent significance in the fully adjusted model (all *p* > 0.05), suggesting that their effects may be mediated or confounded by other variables. These results highlight a distinct profile of both non-modifiable and potentially modifiable factors independently associated with thyroid nodules in this adult health examination population (see Table 3).

**Table 3:** Multivariate logistic regression analysis of independent correlates of thyroid nodules.

Variable	Category	*β*	SE	Wald *χ*^2^	*p* value	OR
Sex (ref: Male)	Female	0.50	0.05	102.45	<0.001	1.65 (1.51–1.80)
Age (per 10-year increment)	Continuous	0.44	0.03	228.67	<0.001	1.55 (1.47–1.64)
Iodine nutrition (ref: Adequate)	Insufficient	0.32	0.06	29.85	<0.001	1.38 (1.23–1.55)
	Excessive	0.16	0.07	5.32	0.021	1.17 (1.02–1.34)
BMI (ref: Normal)	Underweight	0.00	0.12	0.00	0.998	1.00 (0.78–1.29)
	Overweight	0.16	0.04	16.98	<0.001	1.17 (1.08–1.27)
	Obese	0.30	0.05	38.91	<0.001	1.35 (1.21–1.50)
TSH status (ref: Normal)	Abnormal	0.28	0.05	32.67	<0.001	1.32 (1.19–1.46)
TPOAb status (ref: Negative)	Positive	0.25	0.06	17.89	<0.001	1.28 (1.13–1.45)
History of thyroiditis (ref: No)	Yes	0.10	0.07	2.01	0.156	1.11 (0.96–1.28)
Physical activity (ref: <150 min)	≥150 min	−0.32	0.05	43.58	<0.001	0.73 (0.66–0.81)
Family history of thyroid disease (ref: No)	Yes	0.84	0.08	109.32	<0.001	2.31 (2.04–2.61)
Non-neck radiation exposure (ref: No)	Yes	0.16	0.06	8.43	0.004	1.17 (1.05–1.31)
Intercept	–	−1.60	0.14	130.69	<0.001	0.20
Model goodness-of-fit	Hosmer-Lemeshow *χ*^2^ = 8.97, *p* = 0.354; Nagelkerke *R*^2^ = 0.25					

### 3.5 Characteristics and correlates of suspicious malignant nodules

Among the 4,409 participants with sonographically detected thyroid nodules, 176 (4.0%) were classified as having suspicious malignant nodules (TI-RADS 4A or higher). Descriptive analyses revealed notable differences in the profile of these participants compared to those with benign or probably benign nodules. Individuals with suspicious nodules were more frequently aged 50 years or older (58.52% vs. 46.89%), reported a family history of thyroid disease in 28.41% of cases (vs. 8.92%), and exhibited higher rates of thyroid autoimmunity, with TPOAb positivity observed in 26.14% (vs. 11.65%). Furthermore, abnormal TSH levels were more prevalent in this subgroup (29.55% vs. 19.87%). These patterns align with established risk profiles for thyroid malignancy reported in the literature. However, due to the limited number of cases in this category (*n* = 176), a formal multivariable analysis was not performed to control for potential confounders. Therefore, the observed associations should be interpreted as exploratory and hypothesis-generating, highlighting factors that warrant investigation in larger, prospective studies with pathological confirmation. The full comparative profile is presented in Table 4.

**Table 4:** Characteristics of participants with suspicious malignant nodules (TI-RADS 4A+).

Characteristic	Suspicious malignant (*n* = 176)	Benign nodules (*n* = 4,233)	Crude OR (95% CI)	*p* value
Age ≥50 years	103 (58.52)	1,985 (46.89)	1.59 (1.09–2.32)	0.016
Female sex	109 (61.93)	2,690 (63.55)	0.94 (0.64–1.37)	0.752
Family history of thyroid disease	50 (28.41)	376 (8.92)	4.01 (2.65–6.06)	<0.001
Positive TPOAb	46 (26.14)	492 (11.65)	2.63 (1.75–3.96)	<0.001
Abnormal TSH	52 (29.55)	841 (19.87)	1.63 (1.09–2.43)	0.017
Insufficient iodine intake	41 (23.30)	880 (20.79)	1.15 (0.76–1.73)	0.512
Obesity	43 (24.43)	865 (20.43)	1.26 (0.83–1.91)	0.278

## 4 Discussion

This large-scale, cross-sectional study leveraged detailed electronic health records to investigate the epidemiology of thyroid nodules in a health examination population from Jiaozuo, China. By systematically adjusting for key confounders, including iodine nutrition, thyroid function, and autoantibody status, we identified a distinct profile of independent correlates, encompassing both non-modifiable and potentially modifiable factors. The overall nodule prevalence of 35.36% situates this region within the intermediate range reported across China, consistent with the geographic heterogeneity observed in prior studies and likely reflecting regional disparities in iodine nutrition, metabolic profiles, and healthcare access (15–18). Crucially, the vast majority of detected nodules were classified as benign or probably benign, reinforcing the global consensus that most screen-detected nodules are indolent and do not warrant aggressive clinical management. Furthermore, the small number of sonographically suspicious malignant nodules precluded robust multivariate analysis for this subgroup. This finding is thus exploratory and descriptive, highlighting a critical need for future studies with larger, pathologically confirmed samples to identify reliable correlates of malignancy risk.

Our findings confirm the well-established roles of female sex and advancing age as strong, non-modifiable correlates (19). The observed association with female sex may be partly mediated by hormonal influences, particularly estrogen’s role in promoting thyroid tissue proliferation, and the higher prevalence of autoimmune thyroid conditions among women (20). The potent correlation with family history of thyroid disease, the strongest identified in our analysis, underscores the significant contribution of genetic predisposition, potentially involving known susceptibility genes, though shared familial environmental factors likely also contribute (21).

Among modifiable factors, insufficient iodine intake was independently associated with increased nodule risk, a finding that aligns with the pathophysiological understanding that iodine deficiency induces compensatory thyroid hyperplasia (22). Excessive iodine intake was also associated with higher odds, a correlation particularly relevant in this industrial region where local dietary patterns may contribute to sustained above-adequate intake. We hypothesize that in an iodine-replete background, chronic excess iodine may promote nodulogenesis through oxidative stress and sustained proliferative stimuli on thyrocytes (23–26). This proposed mechanism warrants future investigation through studies that integrate detailed dietary surveys with biomarker assessments. The association of obesity with nodules may be explained by underlying metabolic disturbances, such as insulin resistance and elevated levels of insulin-like growth factor-1, which can promote thyrocyte proliferation (27). Similarly, abnormal TSH levels and TPOAb positivity likely contribute to nodulogenesis through direct trophic stimulation and the creation of a chronic inflammatory microenvironment, respectively (28). A key and encouraging finding was the identification of regular moderate-to-vigorous physical activity as a significant protective factor (29). While this association may be partly attributable to broader healthy lifestyle patterns, it nonetheless highlights a promising, feasible target for primary prevention (30). It is noteworthy that several factors significant in univariate analysis, such as smoking and alcohol consumption, were not independently associated in the fully adjusted model, suggesting their apparent effects may be mediated or confounded by other stronger correlates.

This study has several limitations that warrant consideration. Its cross-sectional design precludes causal inference. The single-center, health-examination-based sample may limit generalizability due to potential selection bias, such as the healthy worker effect, whereby participants likely possess greater health awareness and socioeconomic advantages than the general population. This may lead to an underestimation of true prevalence and an attenuation of observed associations for lifestyle factors, suggesting our findings are most directly applicable to similar health-seeking cohorts. Future research directions include incorporating community-based samples and conducting stratified analyses to better delineate the boundaries of these associations. Furthermore, although we incorporated comprehensive laboratory and clinical data, some exposures were subject to measurement bias. Physical activity was based on EHR documentation rather than objective measurement, which likely results in non-differential misclassification and an underestimation of its true protective effect. Subsequent research employing objective accelerometer data and validated questionnaires is planned to verify the robustness of this association. Certain environmental factors relevant to this industrial region were also not available for analysis.

Despite these limitations, our findings carry clear implications for practice. They support the value of targeted thyroid ultrasound screening for high-risk subgroups, including older adults, women, and individuals with a family history of thyroid disease, obesity, or laboratory evidence of iodine insufficiency or thyroid dysfunction. From a public health perspective, these results underscore the potential benefits of community-level interventions aimed at promoting adequate iodine nutrition, maintaining a healthy body weight, and encouraging regular physical activity. For the small proportion of individuals identified with sonographically suspicious nodules, our data reinforce the critical importance of ensuring appropriate referral for cytological evaluation and structured follow-up to exclude malignancy.

## 5 Conclusion

In this EHR-based cross-sectional study, thyroid nodules were prevalent in the Jiaozuo health examination cohort, with most being benign. Female sex, older age, family history of thyroid disease, insufficient iodine intake, obesity, abnormal thyroid function, and non-neck radiation exposure were associated with increased nodule risk, while regular physical activity was a protective factor. These findings provide a basis for region-specific screening and prevention strategies. Future prospective studies with larger, more representative samples are needed to confirm these associations and establish causal relationships.

## Data Availability

The raw data supporting the conclusions of this article will be made available by the authors, without undue reservation.
